# The Impact of Long COVID on the Female Reproductive System: A Narrative Review

**DOI:** 10.3390/ijms27146127

**Published:** 2026-07-09

**Authors:** Ernest Starek, Klaudia Żak, Marta Ostrowska-Leśko, Marcin Bobiński

**Affiliations:** 1 Doctoral School, Medical University of Lublin, 20-059 Lublin, Poland; 2 1st Chair and Department of Gynecologic Oncology and Gynecology, Medical University of Lublin, 20-081 Lublin, Poland; 3 Department of Medical Chemistry, Medical University of Lublin, 20-059 Lublin, Poland; 4 Independent Laboratory of Translational Medicine, Chair of Genetics, Medical University of Lublin, 20-059 Lublin, Poland; marta.ostrowska-lesko@umlub.edu.pl (M.O.-L.); marcin.bobinski@umlub.edu.pl (M.B.)

**Keywords:** long COVID, SARS-CoV-2, female reproductive system, menstrual disturbances, abnormal uterine bleeding, ovarian function, ovarian reserve, hypothalamic-pituitary-ovarian axis, COVID-19 vaccination

## Abstract

Long COVID affects multiple organ systems and disproportionately affects women, raising concerns about reproductive health. This updated narrative review summarizes evidence from PubMed, Scopus, and Web of Science through March 2026 and extends previous reviews by distinguishing Long COVID-specific findings from acute and early post-infection observations and integrating newer evidence on ovarian reserve, follicular-fluid biology, assisted reproduction, and endometrial function. Reported menstrual changes include irregular cycles, heavy or prolonged bleeding, intermenstrual bleeding, and amenorrhea. Evidence regarding ovarian reserve remains inconsistent: some studies reported lower anti-Müllerian hormone (AMH) levels and/or antral follicle count (AFC), whereas others found no significant changes. Case reports describe premature ovarian insufficiency (POI) and possible autoimmune oophoritis but do not establish causality. Proposed mechanisms include immune dysregulation, inflammation, oxidative stress, endothelial dysfunction, and hypothalamic–pituitary–ovarian axis disturbance. Available evidence does not indicate adverse effects of COVID-19 vaccination on ovarian reserve or fertility outcomes. Most studies suggest transient changes. Direct Long COVID-specific evidence for persistent ovarian or endometrial injury remains limited. However, short follow-up precludes conclusions regarding ovarian aging, menopausal timing, lifetime fertility, or long-term endometrial receptivity. Symptom-guided assessment may include menstrual tracking, AMH, early-follicular-phase follicle-stimulating and luteinizing hormones, and pelvic ultrasonography when clinically indicated.

## 1. Introduction

The coronavirus disease 2019 (COVID-19) pandemic, caused by severe acute respiratory syndrome coronavirus 2 (SARS-CoV-2), has raised concerns about its long-lasting health consequences, particularly for the female reproductive system. Recent studies indicate that approximately 10% of infected individuals—approximately 78 million globally —may experience Long COVID (LC), also referred to as post-acute sequelae of SARS-CoV-2 infection (PASC) [1]. Long COVID is generally defined as symptoms occurring usually three months after SARS-CoV-2 infection, lasting for at least two months, and not explained by an alternative diagnosis [2]. The condition is clinically heterogeneous and may involve multiple organ systems, including respiratory, cardiovascular, neurological, endocrine, and reproductive manifestations [3,4,5]. Notably, epidemiological studies indicate that women are at a higher risk of Long COVID than men, with mid-reproductive age groups appearing most vulnerable [6].

At present, Long COVID is diagnosed clinically, primarily based on symptom persistence and exclusion of alternative causes, as no standardized biomarker or diagnostic test has been validated. This distinction is important, as many existing studies compare women with Long COVID to those who only experienced acute COVID-19, which may complicate the interpretation of reproductive outcomes.

Female reproductive function is regulated by the hypothalamic-pituitary-ovarian (HPO) axis and by ovarian follicle health [7]. Even transient disruptions, such as acute illness or psychosocial stress, can perturb menstrual cyclicity and ovulation [8]. Menstrual irregularities have been documented in women with infectious diseases such as human immunodeficiency virus (HIV) [9,10], and anovulation is commonly observed after major infections or stress [11,12]. A limited number of studies have reported persistent menstrual and hormonal disturbances following SARS-CoV-2 infection, including among women with Long COVID, eliciting questions about direct and indirect viral effects on reproductive tissues [13]. This concern was supported by reports that angiotensin-converting enzyme 2 (ACE2), a receptor involved in viral entry, is expressed in the ovaries, uterus, and vagina [14]. However, the available evidence remains heterogeneous, and many early observations were derived from acute or early post-infection cohorts [5,13].

Over five years into the pandemic, significant research has been conducted on the long-term reproductive outcomes following COVID-19. Current research explores ovarian reserve markers (anti-Müllerian hormone [AMH], follicle-stimulating hormone [FSH], luteinizing hormone [LH], and estradiol), menstrual patterns, and assisted reproduction outcomes after SARS-CoV-2 infection [15]. A major concern is whether Long COVID may accelerate ovarian aging or trigger premature ovarian insufficiency (POI), defined as cessation of ovarian function before the age of 40 [16].

Understanding the potential impact of Long COVID on ovarian health and fertility is not only important for individual family planning but also for recognizing broader consequences of PASC as a multisystem disorder. The ovaries act as sensitive indicators of overall health, and any persistent impairment of ovarian reserve in reproductive-age women could have implications for fecundity and menopausal timing, thereby increasing risks of comorbidities associated with estrogen deficiency, such as osteoporosis and cardiovascular diseases [17,18].

Previous reviews have examined female reproductive health in the context of SARS-CoV-2 infection, vaccination, and Long COVID [5,15,19,20,21,22,23]. The present review adds value by updating the evidence through March 2026, explicitly distinguishing Long COVID-specific findings from acute and early post-infection observations, and integrating recent data on ovarian reserve, follicular-fluid biology, assisted reproduction, POI, and endometrial function.

The emphasis on the HPO axis, ovarian reserve, menstrual and ovulatory function, and assisted reproduction reflects the current distribution and measurability of the evidence rather than an assumption that other reproductive organs are unaffected. Because COVID-19 is a systemic disease, the review also considers inflammatory, oxidative, endothelial, immune-mediated, and neuroendocrine pathways, together with emerging evidence concerning the endometrium. Nevertheless, the available follow-up remains too short to determine whether SARS-CoV-2 infection influences ovarian aging, menopausal timing, lifetime fertility, or long-term endometrial receptivity. Reliable assessment of these outcomes will require prospective observation over the next 10–15 years and potentially longer.

The literature was identified through searches of PubMed, Scopus, and Web of Science for publications available through 31 March 2026. Search strings combined exposure-related terms using the Boolean operator “OR” (e.g., “Long COVID”, “post-COVID condition”, and “SARS-CoV-2 infection”) with reproductive outcome terms using “AND” (e.g., menstrual function, ovulation, ovarian reserve, AMH, reproductive hormones, POI, fertility, assisted reproduction, follicular fluid, and endometrial function). Reference lists of relevant studies and reviews were also screened. Priority was given to Long COVID and persistent post-infection outcomes, while selected acute studies were retained for mechanistic or clinical context. Study design, sample size, follow-up, control groups, outcome assessment, and potential bias were considered during interpretation; case reports and small series were treated as hypothesis-generating evidence only.

**Figure 1:**
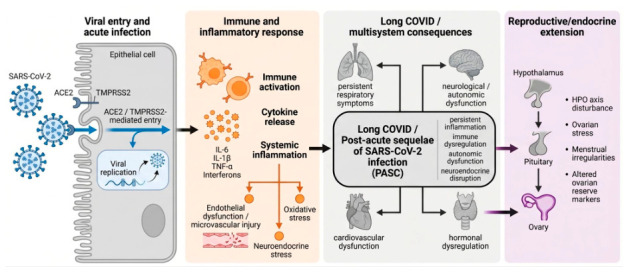
Systemic effects of SARS-CoV-2 infection and Long COVID: from viral entry to reproductive consequences. Created in BioRender. Starek, E. (2026) https://BioRender.com/yvphjub (accessed on 5 July 2026).

**Figure 2:**
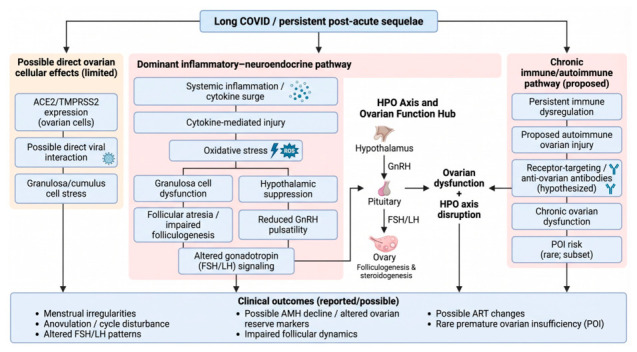
Proposed mechanisms linking Long COVID to ovarian dysfunction and disruption of the hypothalamic–pituitary–ovarian (HPO) axis. Created in BioRender. Starek, E. (2026) https://BioRender.com/a0mkgmq (accessed on 5 July 2026).

Accordingly, this review synthesizes current evidence on menstrual and ovulatory function, ovarian reserve and HPO-axis biomarkers, POI, assisted reproduction outcomes, endometrial involvement, and the proposed mechanisms underlying reproductive alterations in Long COVID. It also identifies major methodological limitations, knowledge gaps, and priorities for future longitudinal research. The systemic effects of SARS-CoV-2 infection and Long COVID, from viral entry to reproductive consequences, are summarized in Figure 1. The proposed mechanisms linking Long COVID to ovarian dysfunction and HPO-axis disruption are summarized in Figure 2.

## 2. Menstrual Cycle and Ovulatory Changes in Long COVID

Multiple studies have documented an association between SARS-CoV-2 infection and subsequent menstrual disturbances, particularly in patients who develop Long COVID. Reported alterations include prolonged cycles (oligomenorrhea), shortened cycles (polymenorrhea), amenorrhea (skipped periods), and changes in bleeding intensity such as menorrhagia or unusually light menses [5,24,25,26,27]. In a large multi-country survey of 1792 menstruating individuals with Long COVID, 36.1% reported new-onset menstrual disturbances after infection [28]. Within this group, 26% experienced irregular cycles, and 19.7% reported heavy bleeding; notably [28], 4.5% of perimenopausal women (>49 years) described unexpected post-menopausal bleeding [28]. Similarly, a Spanish survey of 17,455 women found that 39.4% of them reported menstrual changes since the start of the pandemic, with those identifying as Long COVID patients (*n* = 748) showing higher odds of alterations (adjusted odds ratio ~1.34, 95% CI 1.15–1.57) compared to women without Long COVID [29]. Similar findings were reported in a UK online survey of 12,187 menstruating individuals, where Long COVID (*n* = 1048) was associated with increased abnormal uterine bleeding, including a 93% higher risk of heavier menstrual flow, a 2.26-fold higher prevalence of menses lasting > 8 days, a 59% higher risk of intermenstrual bleeding, and a 39% higher prevalence of missed periods versus never-infected controls (*n* = 9423). These results indicate that Long COVID contributes to menstrual cycle disruption beyond pandemic-related stressors. The potential role of vaccination in modulating menstrual outcomes is still debated. Zhong et al. reported that vaccination may reduce the risk of long-term menstrual disorders associated with SARS-CoV-2 infection [26]. In contrast, Alvergne et al. (*n* = 12,579) found no association between vaccination and abnormal cycle parameters [30]. In the latter study, however, women with prior COVID-19, including those with Long COVID, had increased risks of heavy bleeding, missed periods, and breakthrough bleeding [30].

Menstrual changes in Long COVID often coincide with systemic symptoms. Women reporting both menstrual disturbances and Long COVID were more likely to experience fatigue, headaches, myalgia, and dyspnea [31]. Additionally, Long COVID symptoms often worsen around menstruation: one-third of patients report relapses during or before their period, and up to 62% describe symptom exacerbation in the premenstrual phase [28,32,33]. The findings present a correlation suggesting that Long COVID may adversely affect menstrual health. At the same time, concurrently, hormonal fluctuations may influence Long COVID severity, and Long COVID-related inflammation may disrupt menstrual health.

Ovulatory function is also affected, though findings remain inconsistent [34]. Some studies suggest that stress, inflammation, or hormonal changes in Long COVID may impair ovulation, contributing to menstrual irregularities [8,25]. However, Nguyen et al. and others found no significant increase in anovulatory cycles or altered cycle length compared with pre-pandemic cohorts [35,36,37].

**Figure 3:**
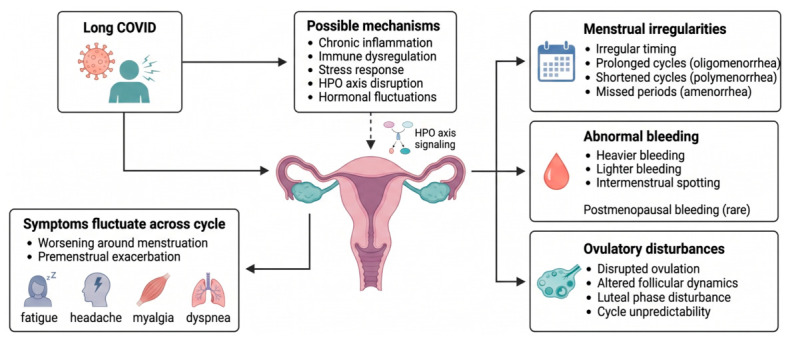
Menstrual cycle and ovulatory changes in Long COVID. Created in BioRender. Starek, E. (2026) https://BioRender.com/sbewcg2 (accessed on 5 July 2026).

Overall, approximately one-third of women with Long COVID report menstrual or ovulatory disturbances. While most irregularities appear transient, a subset of patients experience persistent or clinically significant changes, pointing to the need for long-term follow-up studies. Conflicting findings on vaccination and ovulation emphasize ongoing uncertainties and the influence of multiple interacting factors. These menstrual and ovulatory changes are summarized in Figure 3.

## 3. Ovarian Reserve and Hormonal Biomarkers (AMH, FSH, LH, Estradiol) in Long COVID

One of the central questions has been whether SARS-CoV-2 infection, especially Long COVID, exerts lasting effects on ovarian reserve. Anti-Müllerian hormone (AMH), produced by granulosa cells of early follicles, is a well-established biomarker of ovarian reserve [38]. Early studies suggested a possible decline in AMH following COVID infection. In a Wuhan cohort of 78 women recovered from COVID-19, Ding et al. found significantly lower AMH compared with controls (0.41 ng/mL vs. 1.05 ng/mL) and elevated FSH and prolactin concentrations, with greater decline in women with severe illness [13]. Similarly, Nazari et al. observed reduced AMH levels (2.66 vs. 3.38 ng/mL, *p* = 0.03) in 50 patients with severe disease, and Gullo et al. reported a 27.4% reduction in AMH and fewer antral follicles in 203 women undergoing assisted reproductive technology (ART) after COVID-19 [39,40].

By contrast, other studies did not confirm a significant impact. Li et al. and Wang et al. found no differences in AMH or antral follicle count between recovered women and controls [41,42]. Other researchers also reported no change in AMH before vs. after infection [43].

A 2024 meta-analysis by Ghaemi et al. pooled data from multiple cohorts and found a modest but significant decline in AMH post-COVID (SMD ≈ −0.25), while confirming that vaccination had no effect [20]. A broader 2026 umbrella review published in 2026 by Cao et al., encompassing 14 meta-analyses of fertility and assisted reproduction outcomes, concluded that COVID-19 had minimal overall effects on female ovarian reserve, including AMH and AFC, and on ART outcomes [44]. However, the quality of the underlying evidence was rated as very low to low, and the review was not specific to Long COVID.

Data on Long COVID are scarce. Dogan et al. reported that AMH levels remained significantly reduced six months after recovery, with greater declines associated with more severe illness [45]. In contrast, Maybin et al. found no difference in AMH between women with Long COVID and controls [33].

In summary, findings among the eight primary studies discussed in this section were evenly divided: four reported lower AMH and/or AFC after SARS-CoV-2 infection [13,39,40,45] whereas four found no significant difference compared with control groups or pre-infection measurements [33,41,42,43]. Although the meta-analysis by Ghaemi et al. indicated a small pooled reduction in AMH, the broader umbrella review found minimal overall effects on female ovarian reserve and ART outcomes [20,44]. Neither synthesis specifically addressed well-defined Long COVID cohorts, and the available evidence remains insufficient to determine long-term effects.

Gonadotropin changes have also been noted. FSH and LH measured in the early follicular phase provide insight into the HPO axis feedback [46]. Baglio et al. (2024) conducted a prospective multicenter study of ~200 patients with infertility and pre- and post-infection data, reporting mean increases of ~13.6% in FSH and ~13.4% in LH after COVID-19, with an increased FSH:LH ratio [40]. Other reports were less consistent: Ding et al. described elevated FSH with an increased FSH:LH ratio [13]. At the same time, Madendag et al. and Maybin et al. found no significant change in either gonadotropin [13,33,40,47]. These discrepancies likely reflect differences in disease severity, timing of blood draws, and age distribution of the studied cohorts [13,40,47].

Ding et al. also reported significantly higher prolactin in COVID patients vs. controls (25 vs. 12 ng/mL) [13]. Żak et al. confirmed transient hyperprolactinemia during active infection, with levels returning to baseline within one month and remaining stable thereafter, suggesting no sustained dysregulation in Long COVID [48].

Basal estradiol levels outside the acute phase have not shown consistent changes. Gullo et al. and others noted no difference in mid-follicular estradiol between post-COVID and control groups, whereas women with secondary amenorrhea or POI exhibited frankly low menopausal-range estradiol [40,49].

**Figure 4:**
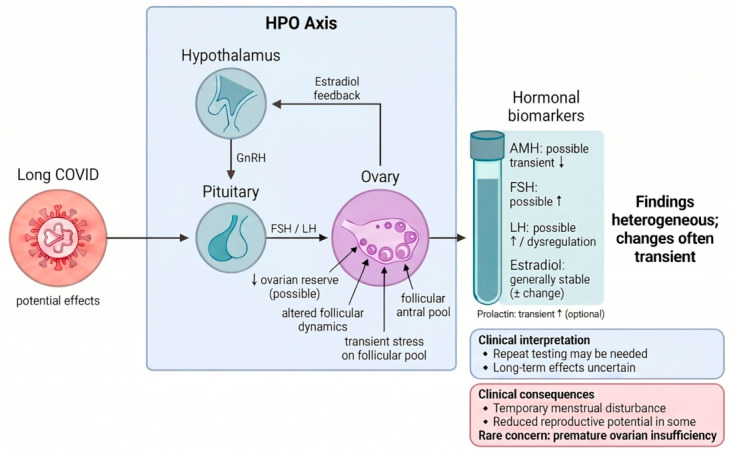
Potential effects of Long COVID on ovarian reserve and hormonal biomarkers. Created in BioRender. Starek, E. (2026) https://BioRender.com/plmsvu5 (accessed on 5 July 2026).

Current evidence suggests that COVID-19 may modestly lower AMH and, in some women, increase FSH and LH, particularly in severe cases. Prolactin appears to rise transiently but normalizes quickly, while estradiol remains largely stable except in women who develop POI. Data directly examining Long COVID remain scarce, demonstrating the need for long-term follow-up studies to clarify whether hormonal changes persist and translate into clinically relevant declines in ovarian reserve. Potential effects on ovarian reserve and hormonal biomarkers are summarized in Figure 4.

## 4. Premature Ovarian Insufficiency (POI) Post-COVID

Premature ovarian insufficiency (POI), defined as loss of ovarian function before the age 40 with amenorrhea, menopausal-range FSH levels, and low estradiol after exclusion of other causes, has been reported in several women following SARS-CoV-2 infection [16]. The first case report by Wilkins and Al-Inizi (2021) described a 35-year-old woman who developed secondary amenorrhea and a menopausal hormonal profile approximately four months after a relatively mild COVID-19 infection, with no prior risk factors or identified alternative explanation [50]. Similar observations were published by Puca et al. (2022), who reported a case of a 27-year-old with eight months of amenorrhea and FSH levels above 75 mIU/mL after infection [49]. More recently, Pankiewicz et al. presented a 36-year-old woman with oligomenorrhea progressing to amenorrhea one month after COVID-19, confirmed POI, FSH 93 mIU/mL, AMH 0.02 ng/mL, small ovaries on ultrasound, and no identifiable autoimmune or genetic cause [17]. Their review summarized 11 additional similar cases worldwide. These reports document a temporal association between SARS-CoV-2 infection and the subsequent diagnosis of ovarian insufficiency but do not establish that infection caused or precipitated POI. An autoimmune mechanism has nevertheless been proposed in some reports.

Gorman et al. (2023) described a single case of transient autoimmune oophoritis presenting as POI after COVID-19 infection, with positive anti-ovarian antibodies and recovery of ovarian responsiveness after high-dose corticosteroid therapy [51]. Stern et al. (2024) subsequently reported three cases of autoimmune premature poor ovarian response after mild or asymptomatic COVID-19, including one case suggestive of autoimmune blockade of the FSH glycoprotein and/or FSH receptor function; ovarian responsiveness improved temporarily during corticosteroid therapy in two patients [52]. Together, these observations raise the hypothesis that post-infectious immune dysregulation may contribute to ovarian autoimmunity. Indeed, several POI cases have other autoimmune findings, such as positive anti-thyroid antibodies, accompanied by post-COVID [52].

**Figure 5:**
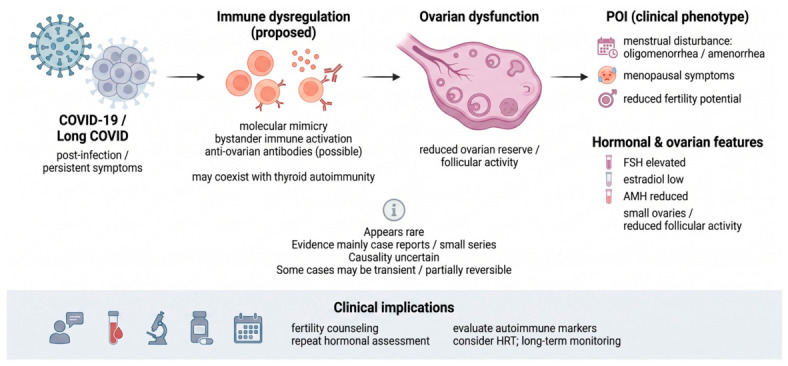
Long COVID premature ovarian insufficiency: proposed mechanisms and clinical features. Created in BioRender. Starek, E. (2026) https://BioRender.com/7h2f5fs (accessed on 5 July 2026).

Although evidence is currently limited to case reports and small series, current evidence is limited to isolated case reports and small case series and cannot establish causality in previously healthy women. Proposed mechanisms include direct ovarian injury and immune-mediated dysfunction. The true incidence is unknown, but the appropriately controlled longitudinal and mechanistic studies are required. Proposed mechanisms and clinical features of post-COVID POI are summarized in Figure 5.

## 5. Follicular Dynamics and Assisted Reproduction Findings

Studies involving women undergoing assisted reproductive technology (ART) offer a unique opportunity to assess follicular dynamics and oocyte competence following COVID-19. Analyses of follicular fluid (FF), oocyte yield, and embryo development may reveal subtle alterations not detected by systemic hormone measurements alone.

Herrero et al. (2022, 2025) showed that anti-SARS-CoV-2 immunoglobulin G (IgG) antibodies are detectable in FF several months after infection, indicating that circulating antibodies can reach the follicular compartment [53,54]. In these studies, higher antibody titers were associated with fewer retrieved oocytes; however, this association does not establish causality. At the same time, cytokine and angiogenic profiling suggested transient alterations in the ovarian microenvironment, including reduced interleukin-1 beta (IL-1β) and initially lower vascular endothelial growth factor (VEGF) levels, but they normalized within 9–18 months. However, the age-stratified analysis suggested a lower number of retrieved mature oocytes, particularly in women older than 36 years. Functionally, FF from recovered women impaired endothelial cell migration in vitro, suggesting transient alterations in the ovarian microenvironment that resolve over time, although clinical significance remains uncertain.

Evidence regarding the impact of prior COVID-19 on ART outcomes remains heterogeneous, and current systematic reviews and meta-analyses do not consistently show a reduction in major in vitro fertilization (IVF) outcomes after mild or asymptomatic prior infection [55,56]. Nevertheless, several cohort studies suggest a short-lived adverse effect when IVF is initiated soon after recovery, particularly within 90 days of infection, with fewer available or top-quality embryos and, in some subgroups, lower oocyte yield [53,57]. Orvieto et al. similarly found no major changes in ovarian stimulation characteristics or overall embryological variables in the first IVF cycle after recovery, apart from a lower proportion of top-quality embryos [58]. In contrast, later age-stratified data suggest that any reduction in ovarian response may be more evident in women older than 36 years [54].

**Figure 6:**
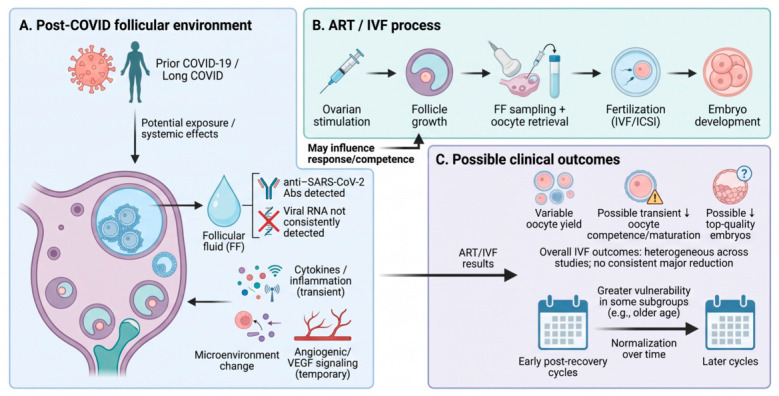
Potential effects of Long COVID on follicular dynamics and ART outcomes. Created in BioRender. Starek, E. (2026) https://BioRender.com/u3g7jaj (accessed on 5 July 2026).

In parallel, ART sampling studies have not demonstrated widespread SARS-CoV-2 ribonucleic acid (RNA) in FF or sampled ovarian or reproductive tissues [53,59,60,61,62]. Instead, the more consistent finding is the presence of anti-SARS-CoV-2 antibodies in FF, which is consistent with follicular immune exposure rather than persistent ovarian infection or ongoing viral replication. However, antibody detection alone does not establish pathogenic follicular injury. Potential effects on follicular dynamics and ART outcomes are summarized in Figure 6.

## 6. Proposed Biological Mechanisms Underlying Female Reproductive Alterations

The link between SARS-CoV-2, the immune system, and ovarian function appears central to the reproductive consequences of Long COVID.

### 6.1. ACE2 Expression and Viral Entry

ACE2, the main receptor for SARS-CoV-2, is expressed in the female reproductive tract, including the ovarian granulosa and cumulus cells [14,63,64,65]. Its expression is hormonally regulated: Choi et al. (2021) found that ACE2 levels in granulosa-lutein cells rise around ovulation under the LH/hCG surge, suggesting a physiological role in follicle rupture and luteal angiogenesis [66]. This upregulation raises the possibility that the peri-ovulatory follicles may be temporarily more susceptible to viral binding; however, ACE2 expression alone does not establish productive infection of ovarian cells [66]. Direct ovarian infection has not been consistently demonstrated. Viral RNA has occasionally been detected in ovarian stromal cells in autopsy studies, but these findings derive from severe or fatal acute COVID-19 and do not establish persistent ovarian infection or Long COVID-specific injury [67,68].

### 6.2. Systemic Inflammation, Oxidative Stress and Cytokine Effects

A cytokine surge, including interleukin-6 (IL-6), interleukin-1 beta (IL-1β), tumor necrosis factor-alpha (TNF-α), and interferons, characterizes severe COVID-19 [69]. These cytokines may disrupt hypothalamic–pituitary signaling, suppress gonadotropin release, and impair estrogen–progesterone feedback [70]. In the ovary, excess of TNF-α and IL-1β may promote granulosa cell apoptosis and accelerate follicular atresia [71]. Oxidative stress, characterized by excessive reactive oxygen species generation and insufficient antioxidant compensation, may promote mitochondrial dysfunction, lipid peroxidation, endothelial injury, and damage to proteins and DNA, thereby impairing granulosa-cell viability, steroidogenesis, oocyte competence, and ovarian microvascular function [72]. Systemic redox imbalance has also been demonstrated in Long COVID: Mese et al. reported increased reactive oxygen metabolites and oxidative stress index in affected patients [73]. Reproductive evidence remains limited; Castiglione Morelli et al. identified altered redox-related and inflammatory profiles in follicular fluid after SARS-CoV-2 infection, including changes in antioxidant enzymes, suggesting compensatory redox responses rather than established persistent oxidative ovarian injury [74]. A systematic review by Voros et al. (2025) identified oxidative stress and systemic inflammation as plausible mechanisms of post-COVID ovarian dysfunction; however, their long-term relevance, particularly in well-defined Long COVID cohorts, remains uncertain [19].

### 6.3. Autoimmune Oophoritis

Viral infections are known triggers of autoimmunity [75,76], and SARS-CoV-2 has been associated with new-onset autoimmune disorders such as type 1 diabetes, lupus, and thyroiditis [51]. The ovary can similarly become a target. Autoimmune oophoritis, a rare but recognized cause of POI, is characterized by antibodies and T-cell responses against ovarian antigens [77]. Case reports following COVID-19 have described transient POI with detectable anti-ovarian antibodies, raising the possibility of molecular mimicry and immune cross-reactivity [51]. Computational assessments have identified sequence overlaps between SARS-CoV-2 proteins and human reproductive proteins, and it is also possible that inflammatory injury may expose normally sequestered ovarian antigens, triggering autoimmunity [78,79]. However, sequence homology does not establish clinically relevant molecular mimicry or autoimmune ovarian injury.

In some women with POI reported after COVID-19, concurrent autoimmune markers, including thyroid autoimmunity, were also described [51]. Ovarian responsiveness improved temporarily during corticosteroid therapy in isolated cases; however, these observations cannot distinguish a treatment effect from spontaneous fluctuation or other confounding factors [52]. These findings should therefore be regarded as hypothesis-generating and do not establish that SARS-CoV-2 causes autoimmune oophoritis.

### 6.4. Hypothalamic-Pituitary Effects

**Figure 7:**
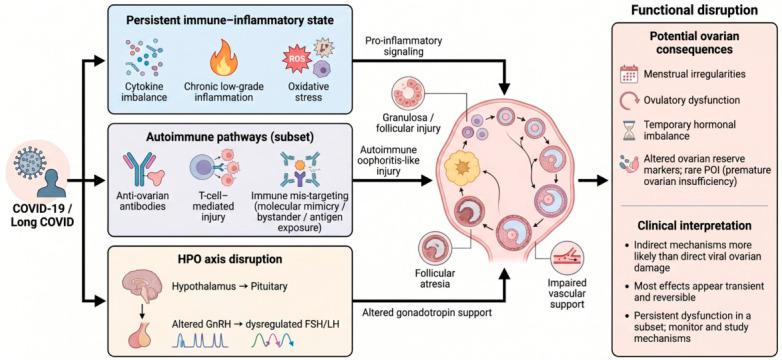
Proposed autoimmune and inflammatory mechanisms linking Long COVID to ovarian dysfunction. Created in BioRender. Starek, E. (2026) https://BioRender.com/mhkyg2v (accessed on 5 July 2026).

The HPO axis may also be disrupted centrally. ACE2 expression has been reported in hypothalamic and pituitary tissues, and viral RNA has been detected in these regions at autopsy [80]. However, direct evidence of persistent hypothalamic–pituitary infection or dysfunction in women with Long COVID remains limited. Acute systemic inflammation and physiological stress response can suppress GnRH pulsatility, leading to low gonadotropins and anovulation—a state similar to functional hypothalamic amenorrhea [81]. While Long COVID hormone profiles often show elevated FSH (indicating primary ovarian dysfunction), some menopausal-like symptoms in Long COVID could stem from central dysregulation or autonomic dysfunction. Estrogen deficiency itself may exacerbate inflammation, whereas normal estrogen levels have protective, immunomodulatory roles, suggesting a potential bidirectional interaction between hormonal status and Long COVID severity [82]. The proposed autoimmune, inflammatory, and HPO-axis mechanisms are summarized in Figure 7.

### 6.5. Endometrial Susceptibility, Receptivity, and the Broader Reproductive Tract

Although the available literature has focused predominantly on ovarian and menstrual outcomes, potential effects on the endometrium and other reproductive tissues should also be considered. A single-cell transcriptomic analysis of 73,181 endometrial cells from 27 donors demonstrated low and heterogeneous expression of canonical SARS-CoV-2 entry factors [83]. Co-expression of ACE2 and TMPRSS2 was identified in only 0.07% of luminal epithelial cells during the mid-secretory implantation window, suggesting a low probability of direct endometrial infection through the canonical entry pathway [83]. These findings should not be interpreted as the complete absence of ACE2 from the endometrium. A protein-level study detected ACE2 in endometrial epithelial and stromal cells, with increased stromal expression during the secretory phase and decidualization; experimental ACE2 knockdown impaired the decidualization response in human endometrial stromal cells [84]. Small preliminary studies also failed to detect SARS-CoV-2 RNA in endometrial biopsies or endometrial tissue samples from women with COVID-19, although these findings do not exclude transient infection or indirect tissue dysfunction [61,85].

**Figure 8:**
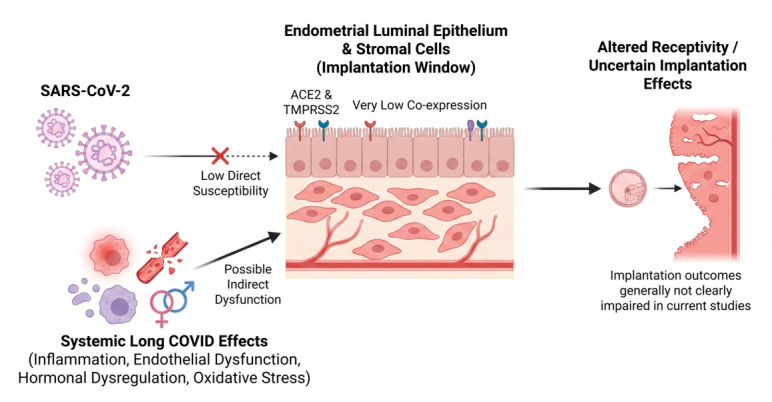
SARS-CoV-2 and the endometrium: direct and indirect effects. Created in BioRender. Starek, E. (2026) https://BioRender.com/6pwe6js (accessed on 5 July 2026).

Accordingly, the direct susceptibility of the endometrium to SARS-CoV-2 remains uncertain and may differ according to menstrual-cycle phase, the viral entry pathway, and the method used to assess receptor expression. Available assisted reproduction studies and meta-analyses have not consistently demonstrated major impairment of implantation or pregnancy outcomes after SARS-CoV-2 infection [55,56,86]. However, most studies assessed relatively short-term ART outcomes and were not designed to detect subtle molecular changes or delayed Long COVID-specific effects on endometrial receptivity. Systemic inflammation, endothelial dysfunction, hormonal dysregulation, and oxidative stress could theoretically affect decidualization and embryo–endometrial interactions even in the absence of persistent endometrial infection [84,87]. Longitudinal studies incorporating molecular receptivity markers, implantation, pregnancy loss, and live-birth outcomes are therefore required. Direct and indirect endometrial effects are summarized in Figure 8.

## 7. Overall Synthesis and Clinical Implications

### 7.1. Overall Interpretation of the Evidence

This review presents a comprehensive synthesis of current evidence regarding the impact of Long COVID on female reproductive health. By critically integrating findings from menstrual cycle studies, hormonal biomarkers, assisted reproduction outcomes, and mechanistic research, a complex but coherent picture emerges: SARS-CoV-2 infection can transiently disrupt ovarian physiology in a small subset of women [5,19,20,52]. Long COVID influences female reproductive health through multiple pathways, including menstrual irregularities, transient hormonal fluctuations, and, in rare cases, premature ovarian insufficiency (POI), although causality and the role of immune-mediated mechanisms remain uncertain [5,17,20,28]. While most effects appear reversible, the persistence of disturbances in some women points to the need for persistent vigilance and further research [20,27,45,54].

Long COVID is consistently associated with menstrual irregularities, typically affecting about one-third of women [5,28,29]. These conclusions parallel other systemic illnesses where stress and inflammation disrupt cyclicity [8,9,10,11]. In most cases, cycles normalize within months, but a minority experience prolonged or clinically significant changes, sometimes requiring medical intervention [5,27,30]. The key clinical implication is that menstrual history should be actively assessed in women with Long COVID, both as a marker of reproductive health and as a potential indicator of broader systemic involvement [5,27,28].

Initial reports of reduced AMH and elevated FSH raised concern about accelerated ovarian aging post-COVID [13,39,40]. More recent studies and meta-analyses suggest that these effects are modest and largely reversible, though women with severe infections may be more vulnerable [20,33,45,47]. Importantly, Long COVID-specific data remain scarce [5,33]. The prevailing interpretation is that SARS-CoV-2 infection temporarily stresses the follicular pool and HPO axis rather than causing irreversible loss of ovarian reserve [13,45,47,54]. Clinicians should be cautious in interpreting abnormal hormone results obtained shortly after infection and consider repeat testing before concluding long-term fertility potential [45,46,47,48].

Case reports have described POI after COVID-19, with autoimmune oophoritis proposed as a possible but unconfirmed mechanism [17,50,51,52]. Although causality cannot be definitively proven, these observations raise the possibility of post-infectious ovarian dysfunction in susceptible individuals but do not establish that SARS-CoV-2 precipitates ovarian failure [17,50,51,52]. While uncommon, even a very low incidence translates to significant numbers at the population level, drawing attention to the importance of monitoring reproductive function after COVID-19 and ensuring standard diagnostic evaluation for alternative causes of amenorrhea or confirmed POI, including autoimmune and endocrine disorders when clinically indicated [17,51,52]. Evidence points to indirect pathways—systemic inflammation, oxidative stress, and immune dysregulation—rather than widespread direct viral invasion of the ovary [53,59,62,67,68]. Autoimmune mechanisms have been proposed on the basis of isolated reports of anti-ovarian antibodies and steroid-responsive ovarian dysfunction, although the evidence remains limited [51,52,75]. Central disruption of the hypothalamic–pituitary axis may also contribute, though most hormonal profiles suggest primary ovarian rather than central dysfunction [13,70,80,81]. A bidirectional interaction is likely: ovarian injury lowers estrogen, which may, in turn, exacerbate the inflammatory state of Long COVID, perpetuating symptoms [32,82].

Beyond ovarian effects, inflammatory, endothelial, and redox disturbances could also influence endometrial function and decidualization, although direct Long COVID-specific evidence remains limited [84,87].

This review highlights several key insights. First, Long COVID is not only a systemic condition but also presents specific reproductive signatures, including menstrual irregularities, altered ovarian reserve markers, and rare but serious cases of autoimmune oophoritis [5,20,28,52]. Second, most reproductive effects appear reversible, which provides reassurance to patients and clinicians while also identifying a minority of women at risk of lasting harm [17,20,27,45]. Third, potential therapeutic windows are identified: early recognition of POI, monitoring for autoimmune markers, and precise interventions may help preserve fertility and hormonal health in vulnerable patients, while specific immune-targeted interventions cannot yet be recommended. Finally, current evidence confirms that vaccination does not adversely affect ovarian function and may indirectly protect reproductive health by preventing severe disease [1,20,30,54,59].

### 7.2. Clinical Implications

For most women, COVID-19 is unlikely to cause permanent infertility, but awareness of transient reproductive effects is important for counseling and care [15,55,56]. In clinical practice, menstrual changes and hormonal testing may provide useful insight into Long COVID patients with persistent symptoms or fertility concerns [5,27,46]. Early recognition of POI allows timely initiation of hormone replacement therapy and fertility counseling [16,17]. Importantly, evidence consistently shows that COVID-19 vaccination does not impair ovarian reserve or fertility outcomes, delivering reassurance to patients and pointing to the value of prevention [20,30,54,57,59].

In women with persistent amenorrhea, abnormal uterine bleeding, menopausal symptoms, or fertility concerns, symptom-guided assessment may include AMH, early-follicular-phase FSH, LH, and estradiol, together with pelvic ultrasonography when clinically indicated. No endometrial receptivity or oxidative-stress biomarker can currently be recommended for routine clinical use in women with Long COVID.

### 7.3. Limitations of the Available Evidence and This Review

The available evidence is heterogeneous and methodologically limited. Studies differed in design, follow-up timing, and definitions of Long COVID, and many were small, retrospective, cross-sectional, or based on selected samples and self-reported outcomes [5,15,19,55,56]. Pre-infection measurements, matched control groups, and adjustment for relevant confounders were often unavailable. Several studies evaluated acute or early post-infection outcomes rather than well-defined Long COVID cohorts, while assisted reproduction studies, although providing objective data, involved selected infertility populations. Distinguishing biological effects of SARS-CoV-2 from pandemic-related stressors remains difficult [8,31,36,37], the role of estrogen is uncertain [6,32], and it is unclear whether reproductive alterations are specific to Long COVID or may follow other systemic viral illnesses. Evidence concerning the endometrium and other non-ovarian reproductive tissues remains limited and derives largely from transcriptomic, receptor-expression, and small preliminary clinical studies [83,84,85]. Direct human evidence linking oxidative stress and immune-mediated pathways to persistent reproductive dysfunction is also scarce [74].

Evidence concerning premature ovarian insufficiency and autoimmune oophoritis is based mainly on isolated case reports and small case series and therefore cannot establish incidence, excess risk, or causality. The short duration of follow-up also precludes conclusions regarding ovarian aging, menopausal timing, lifetime fertility, or long-term endometrial receptivity; these outcomes will require prospective observation over the next 10–15 years and potentially longer.

### 7.4. Future Research Directions

Robust prospective, longitudinal, and preferably multicenter cohorts are needed to determine whether Long COVID influences ovarian aging, menopausal timing, or long-term fertility. Such studies should combine repeated assessments of reproductive function with serial systemic and tissue-specific markers of inflammation, oxidative stress, and redox balance and should extend beyond ovarian endpoints to include endometrial receptivity, decidualization, implantation, pregnancy loss, and live-birth outcomes. Analyses should be stratified by age at infection, Long COVID phenotype, symptom severity and duration, vaccination status, baseline ovarian reserve, and pre-existing reproductive disorders. Reliable evaluation of ovarian aging, lifetime fertility, and long-term endometrial function will require follow-up over the next 10–15 years and potentially longer. Mechanistic studies should further investigate autoimmune, inflammatory, oxidative, endothelial, mitochondrial, and neuroendocrine pathways and identify potential biomarkers of reproductive involvement. Interventional studies evaluating anti-inflammatory, immunomodulatory, or hormonal approaches should be undertaken only after causal mechanisms and clinically relevant target populations have been more clearly defined.

## 8. Conclusions

In conclusion, Long COVID presents a novel challenge to women’s reproductive health. Although the ovaries and menstrual cycles generally demonstrate resilience, the possibility of accelerated ovarian aging or immune-mediated ovarian failure remains a concern. Recognizing and addressing these risks is essential for both individual fertility and public health, given the millions of women worldwide affected by Long COVID. This review provides a foundation for clinicians and researchers to ensure that reproductive health is considered in efforts to understand and mitigate the long-term consequences of COVID-19.

## Data Availability

No new data were created or analyzed in this study. Data sharing is not applicable to this article.

## Abbreviations

The following abbreviations are used in this manuscript:

ACE2	Angiotensin-converting enzyme 2
AMH	Anti-Müllerian hormone
ART	Assisted reproductive technology
COVID-19	Coronavirus disease 2019
FF	Follicular fluid
FSH	Follicle-stimulating hormone
GnRH	Gonadotropin-releasing hormone
hCG	Human chorionic gonadotropin
HIV	Human immunodeficiency virus
HPO	Hypothalamic-pituitary-ovarian
IL-1β	Interleukin 1 beta
IL-6	Interleukin 6
IVF	In vitro fertilization
LC	Long COVID
LH	Luteinizing hormone
PASC	Post-acute sequelae of SARS-CoV-2 infection
POI	Premature ovarian insufficiency
RNA	Ribonucleic acid
SARS-CoV-2	Severe acute respiratory syndrome coronavirus 2
TNF-α	Tumor necrosis factor alpha
VEGF	Vascular endothelial growth factor
